# Agent-based modeling of health resources for older adults: accessibility, equity, and last-mile solutions in Fuzhou, China

**DOI:** 10.3389/fpubh.2025.1698911

**Published:** 2025-11-24

**Authors:** Qiuyi Zhang, Xin Wu, Siying Wu, Liyun Huang, Chong Peng

**Affiliations:** 1 College of Architecture and Urban Planning, Fujian University of Technology, Fuzhou, China; 2 Key Laboratory of New Technology for Construction of Cities in Mountain Area, Ministry of Education, Chongqing University, Chongqing, China

**Keywords:** agent-based modeling, older adults health equity, spatial accessibility, urban health planning, Fuzhou’s main urban area

## Abstract

Population aging presents a critical challenge for urban sustainability and health equity. This study addresses the structural mismatch between the spatial distribution of older adults and health resources in Fuzhou, China, by developing an Agent-Based Model (ABM) to simulate the utilization of prevention, treatment, and long-term care facilities and its impact on health outcomes. The model, grounded in empirical questionnaire data and real-world spatial data, incorporates older adults as mobile agents with diverse health, economic, and living statuses, navigating a realistic urban environment over a 500-day simulation. This research designed a conceptual framework capturing the dynamic feedback between facility usage and health status. Four intervention scenarios, Multi-functional Community Centers (S1), Senior-Friendly Transportation Policies (S2), Community-based Health Education (S3), and a Comprehensive package (S4), were tested. Results indicate that spatial accessibility is the paramount driver of health improvement, with S2 (transport support) and S4 (comprehensive) demonstrating the most significant and rapid gains. Health equity, assessed via a weighted Gini coefficient, showed that while S4 achieved the most robust equity improvements across health and economic strata, its effect on residential disparities was limited. S1 risked exacerbating inequities if not precisely targeted, and S3 showed the least efficacy. The study concludes that optimizing health resources for aging populations requires a spatially-anchored, multi-dimensional strategy that prioritizes transportation accessibility, precision facility siting, and integrated service provision to effectively overcome “last-mile” barriers and address the complex needs of socioeconomically diverse older adults.

## 1 Introduction

Population aging has become a defining global challenge of the 21st century, profoundly reshaping societal development trajectories worldwide (1). As the developing country undergoing the most rapid and extensive demographic aging, China is compelled to evolve from providing basic support for older adults to ensuring high-quality aged care, a crucial objective in modernizing national governance (2). The Outline of the Healthy China 2030 Initiative identifies older adults as a priority group and explicitly calls for establishing an integrated health security system that spans prevention, diagnosis, treatment, rehabilitation, and long-term care (3). Achieving comprehensive health security throughout later life requires optimizing the allocation of health resources for older adults across prevention, treatment, and supportive care dimensions, thereby addressing the “last-mile” delivery challenges in health services (4).

Health resources for older adults encompass urban resources with social or natural attributes that promote physical and mental well-being in daily life. These include park spaces, medical institutions, and older adults care facilities, which older adults utilize either actively or passively to maintain health (5). Research on allocating these resources is grounded in public service distribution theory, often focusing on single-type facilities while incorporating the dual perspectives of “older adults” and “health resources” (6). Such studies span allocation standards (7), spatial distribution (8), evaluation and optimization (9), and service performance (10, 11). For instance, spatial equity of community-based care facilities in Shanghai was assessed using Gini coefficients and Lorenz curves (12), while embedded care facilities in Tianjin were shown to significantly improve life satisfaction through social and environmental factors (13). Recent behavioral studies have further explored how daily activities influence health, guiding contextualized facility planning (14). Examples include SEM modeling of park features affecting health behaviors in Zhengzhou (15) and machine learning analyses of health behaviors among ethnic older adults in rural Hunan (16).

Despite these advances, current research overly emphasizes spatial layout and service efficiency, paying insufficient attention to health outcomes (17), such as chronic disease intervention, and offering incomplete theoretical-practical frameworks for integrating full-chain health security into resource planning (18, 19). Crucially, health equity remains underexplored (20–22), particularly regarding systematic disparities in health opportunities and resource accessibility influenced by income, location, and baseline health (23, 24). In terms of methodological approaches to health equity assessment, a systematic comparison of various measurement tools, including the Gini coefficient and concentration index, has highlighted their respective applicability and limitations (25). Furthermore, growing attention has been directed toward the health effects of policy interventions. For instance, rigorous causal inference analyses have demonstrated that clean heating policies significantly reduced the incidence of respiratory diseases among residents. This finding not only provides empirical support for the synergistic optimization of environmental and health resources, but also underscores the necessity of multidimensional health intervention strategies. The observed disparities in the distribution of health benefits across different groups further offer critical evidence for evaluating the equity impacts of public policies (26).

Agent-Based Modeling (ABM) presents a promising approach to address these gaps (27, 28). ABM enables multi-scale simulation of human-environment interactions with minimal data input and supports low-cost, multi-scenario testing for spatial strategy optimization (29, 30). Its key strength lies in dynamically integrating behavioral theories with spatial contexts to simulate complex systems, thereby supporting empirical and applied studies (31). For instance, in a health policy application, a multi-theory ABM simulated health-seeking behaviors and diagnostic delays among diabetes patients. Scenario analyses demonstrated that targeted interventions, such as gender-specific health campaigns or improved primary care access, could substantially reduce late diagnosis rates, offering both theoretical insights and practical decision support for regional disease control strategies (32). Although ABM has been applied to simulate outdoor activities of older adults in Shenzhen (33) and demand-response in community care centers (34), few studies model the dynamic feedback between individual behaviors, such as facility use, and long-term health outcomes (35).

This study aims to bridge this gap by employing ABM, combined with questionnaire surveys and spatial analysis, to simulate health outcomes resulting from older adults’ utilization of prevention, treatment, and long-term care facilities in urban Fuzhou. It conceptualizes the “last mile” as the composite of bottlenecks occurring at the final stage of health resource access for older adults (36, 37). While recognizing the multidimensional nature of these barriers, the study operationalizes the concept by focusing specifically on spatial accessibility, thereby transforming a broad policy metaphor into a measurable and intervenable variable within the model, in alignment with China’s national aging strategy goals (2, 5, 38).

By incorporating bidirectional feedback between facility usage and health status, this study evaluate health impacts and equity under four intervention scenarios, offering spatiotemporally adaptive pathways for optimizing health resource allocation for older adults. The four intervention scenarios include: (1) Multi-functional Community Centers (S1): Upgrading community centers into comprehensive health hubs to provide daycare, preventive screenings, and social services; (2) Senior-Friendly Transportation Policies (S2): Overcoming travel challenge through health-dedicated lines, barrier-free bus retrofits, and pedestrian environment improvements; (3) Community-based Health Education Activities (S3): Conducting health lectures and family workshops to enhance chronic disease self-management skills and preventive healthcare awareness; (4) Comprehensive Intervention Package (S4): Integrating the aforementioned three strategies to test multi-dimensional synergistic effects. All scenarios are based on empirical data, aiming to improve the accessibility of health resources from spatial, social, and cognitive dimensions.

## 2 Study context

### 2.1 Study area

**Figure 1:**
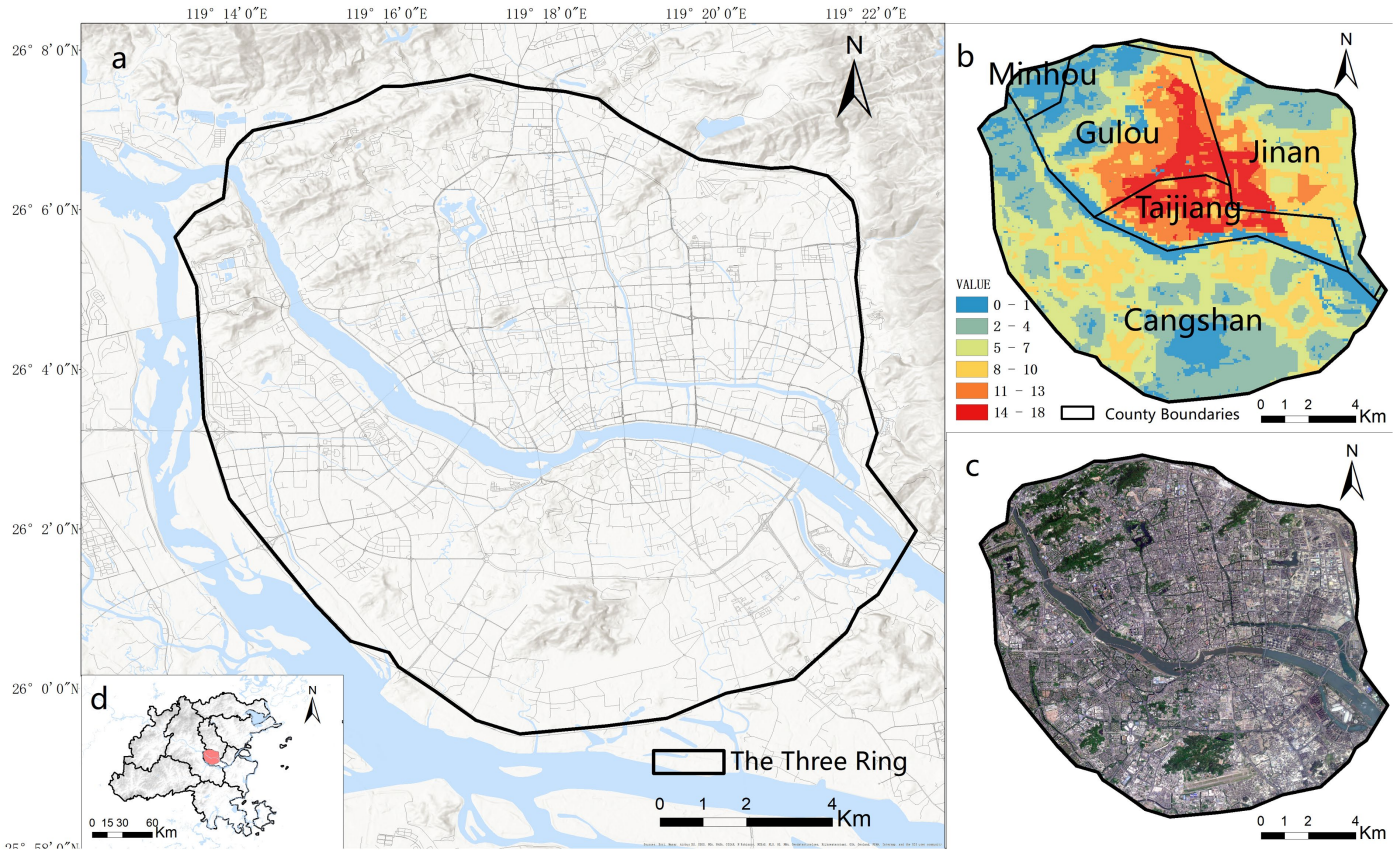
Location and overview of the study area. **(a)** Study area boundary; **(b)** spatial distribution of older adult population density; **(c)** satellite imagery; **(d)** geographical context of the study area within Fuzhou City. Figure 1 was generated by Esri ArcGIS 10.7 online platform (https://www.esri.com/zh-cn/arcgis/products/arcgis-desktop/overview). Sources: Esri, Maxar, Airbus DS, USGS, NGA, NASA, CGIAR, N Robinson, NCEAS, NLS, OS, NMA, Geodatastyrelsen, Rijkswaterstaat, GSA, Geoland, FEMA, Intermap, and the GIS user community.

The study focuses on the central urban area of Fuzhou within the Third Ring Road (Figure 1). As the capital of Fujian Province and a key city in China’s economically dynamic eastern coastal region, Fuzhou serves as the political, economic, cultural, and transportation core of both the city and the province. The study area encompasses most of Gulou, Taijiang, Cangshan, and Jin’an districts, along with part of Minhou County, forming the most densely populated and highly urbanized zone in the city, with a total area of approximately 174.21 km^2^. According to the Seventh National Population Census, Fuzhou has a permanent population of 8.29 million, including 1.38 million people aged 60 and above, indicating an aging rate of 16.76% (39). Fuzhou exhibits urban characteristics, such as rapid urbanization, demographic transition, and ongoing spatial restructuring, that are representative of many Chinese cities (40). Its main urban area within the Third Ring Road constitutes a typical high-density urban core with a concentrated population distribution, making it a highly relevant context for examining accessibility challenges related to health resources for older adults (27, 41).

The Fujian provincial guidelines for urban and rural older adults care facility planning and allocation proposed early guidance for tiered allocation of older adults’ care facilities across three dimensions: residential care, medical-nursing services, and recreational-cultural activities (42). As a typical aging city in Fujian, Fuzhou has implemented systematic policy adjustments for optimizing older adults health resources. The implementation plan for establishing a comprehensive older adults health service system in Fuzhou identifies preventive healthcare, disease diagnosis/treatment, and rehabilitation nursing as core tasks for building this system (43).

### 2.2 Conceptual framework of facility usage and health effects

**Figure 2:**
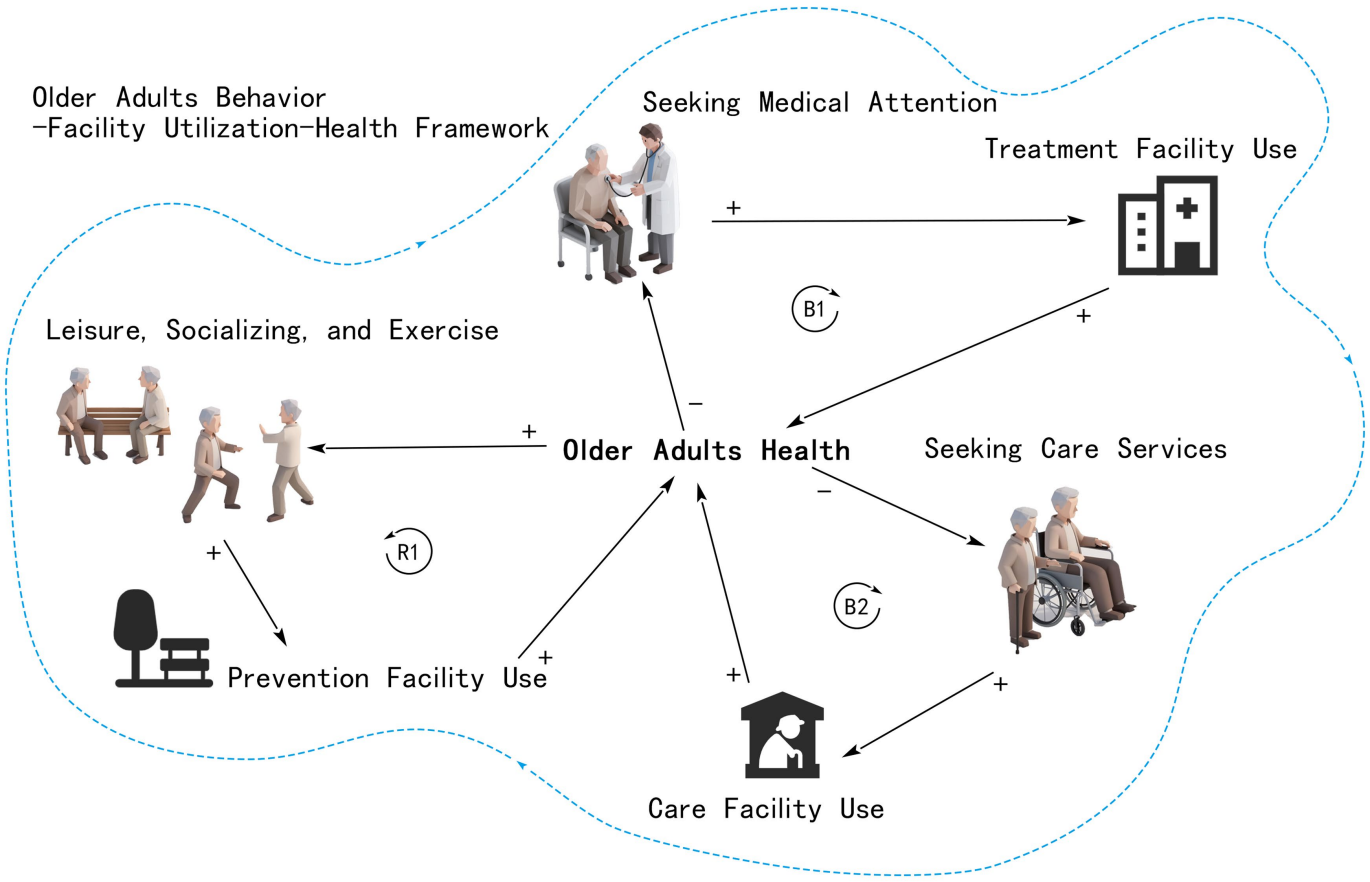
Conceptual framework of the feedback mechanisms between older adults’ facility usage and health outcomes. The icons and figure elements were generated using the AI image generation tool from iconfont (https://www.iconfont.cn/), and subsequently modified by the authors.

Older adults, as a key target of health interventions, typically engage in limited daily activities, primarily involving physical and social interactions, medical visits, and rehabilitative care. Prevention, treatment, and long-term care facilities serve as spatial anchors for these activities, forming an integrated health promotion model that combines spatial resources and behavioral patterns to achieve closed-loop health interventions (44, 45). This study constructs a conceptual framework (Figure 2) to illustrate the dynamic relationships among older adults’ behaviors, facility usage, and health outcomes. Here, “+” denotes positive feedback that enhances health, while “–” indicates negative feedback that reduces demand. The framework incorporates three feedback mechanisms: (1) Reinforcing Loop R1: social and physical activities promote the use of preventive facilities, improving physical and mental health, which in turn encourages further activity engagement; (2) Balancing Loop B1: medical-seeking behavior leads to treatment, which improves health and reduces subsequent demand for medical services; and (3) Balancing Loop B2: use of rehabilitative care facilities aids recovery, decreasing the need for further care.

### 2.3 Data sources and calibration

Population data for adults aged 60 and above were sourced from WorldPop, utilizing a version calibrated with United Nations population estimates (46) and cross-validated against China’s Seventh National Population Census. Socioeconomic and health-related variables were derived from the Fuzhou population census and further supplemented and calibrated through a structured questionnaire survey.

The questionnaire data were collected using a multi-stage stratified sampling approach conducted on-site across Fuzhou’s main urban area. Sampling strata were defined by types of high-frequency activity venues commonly used by older adults, including parks and plazas, medical institutions, and older adults care facilities. A total of 350 questionnaires were distributed to adults aged 60 and above, resulting in 326 valid responses. The survey instrument captured key variables such as socioeconomic attributes, self-rated health status, travel behavior (e.g., outing frequency and travel distance), and facility usage preferences (see Appendix A1 for details). To ensure data reliability, portions of the survey responses were cross-validated with official census records, thereby strengthening sample representativeness and enhancing response validity.

Location data for public service facilities, initially classified under four major categories and eight subcategories, were acquired through the Amap POI (Point of Interest) service (https://www.amap.com). From this dataset, POIs were selected representing three key health-related functions - prevention, including parks and squares; treatment, including various medical institutions; and care, including older adults care service facilities.

## 3 Research methodology

The model was developed following the ODD (Overview, Design Concepts, and Details) protocol, a standardized framework for describing Agent-Based Models (ABM), which systematically outlines model structure, agent design, and operational mechanisms (47). To better capture behavioral complexity, the ODD+D extension was adopted, incorporating explicit descriptions of decision-making processes. The model was implemented using AnyLogic 8.7 Professional, leveraging its GIS capabilities to simulate agent mobility over real-road networks and to configure behavioral rules via parameter settings and Java coding.

### 3.1 Overview

#### 3.1.1 Purpose

**Figure 3:**
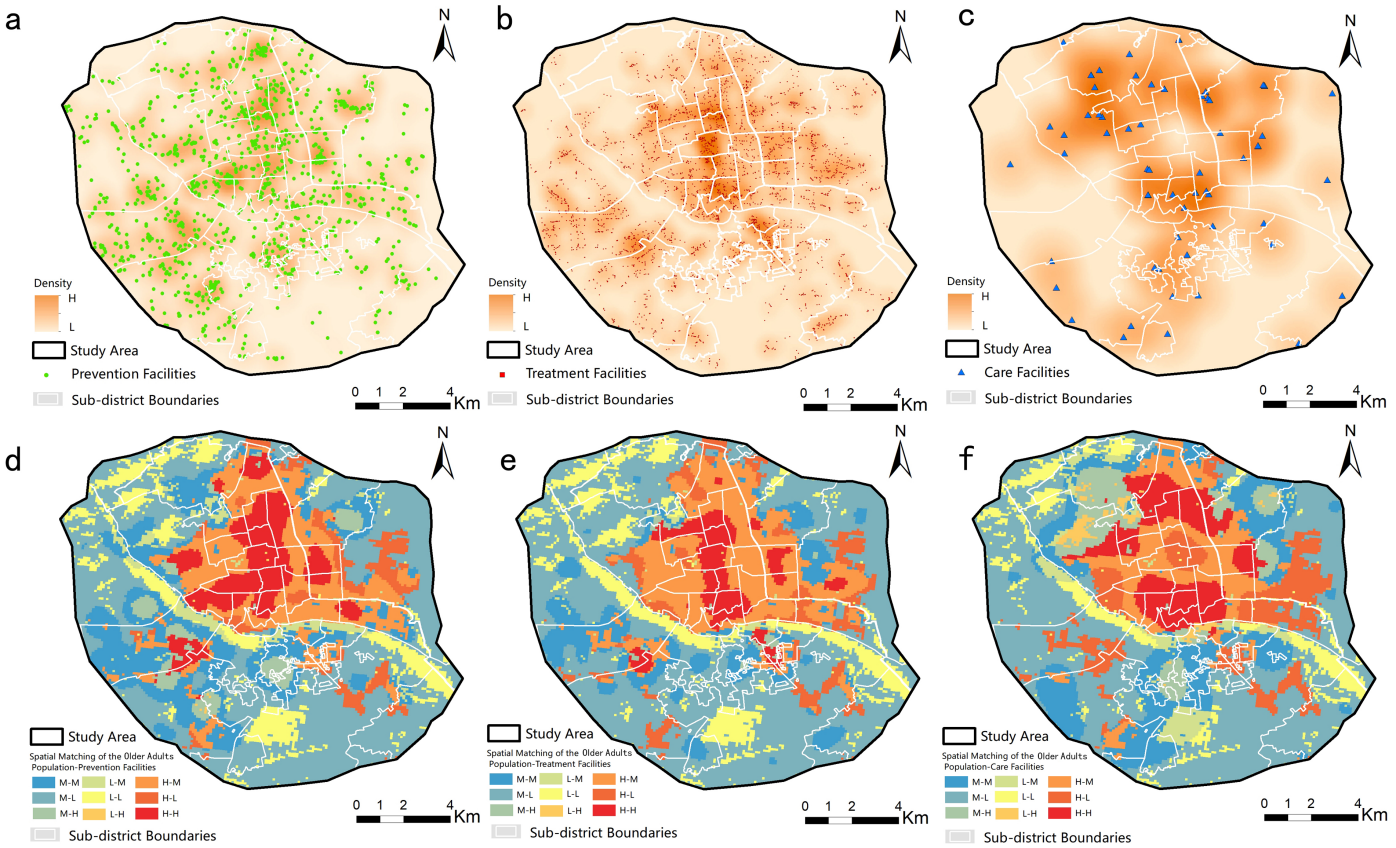
Spatial patterns and correlation analysis of health resources. Notes: Kernel density distribution of **(a)** prevention, **(b)** treatment, and **(c)** long-term care facilities. Spatial correlation between older adult population density and **(d)** prevention, **(e)** treatment, and **(f)** long-term care resources. Maps created with ArcGIS 10.7 (https://www.esri.com/zh-cn/arcgis/products/arcgis-desktop/overview).

The model aims to analyze the health impacts of prevention, treatment, and long-term care facility usage among older adults with varying economic and living conditions, and to assess how interventions affect health outcomes and equity. It addresses demonstrated mismatches between the spatial distribution of older adluts populations and health resources in Fuzhou (Figure 3), where existing policies have yet to resolve persistent inequities (48).

#### 3.1.2 Entities, state variables and scales

##### 3.1.2.1 The environment

**Figure 4:**
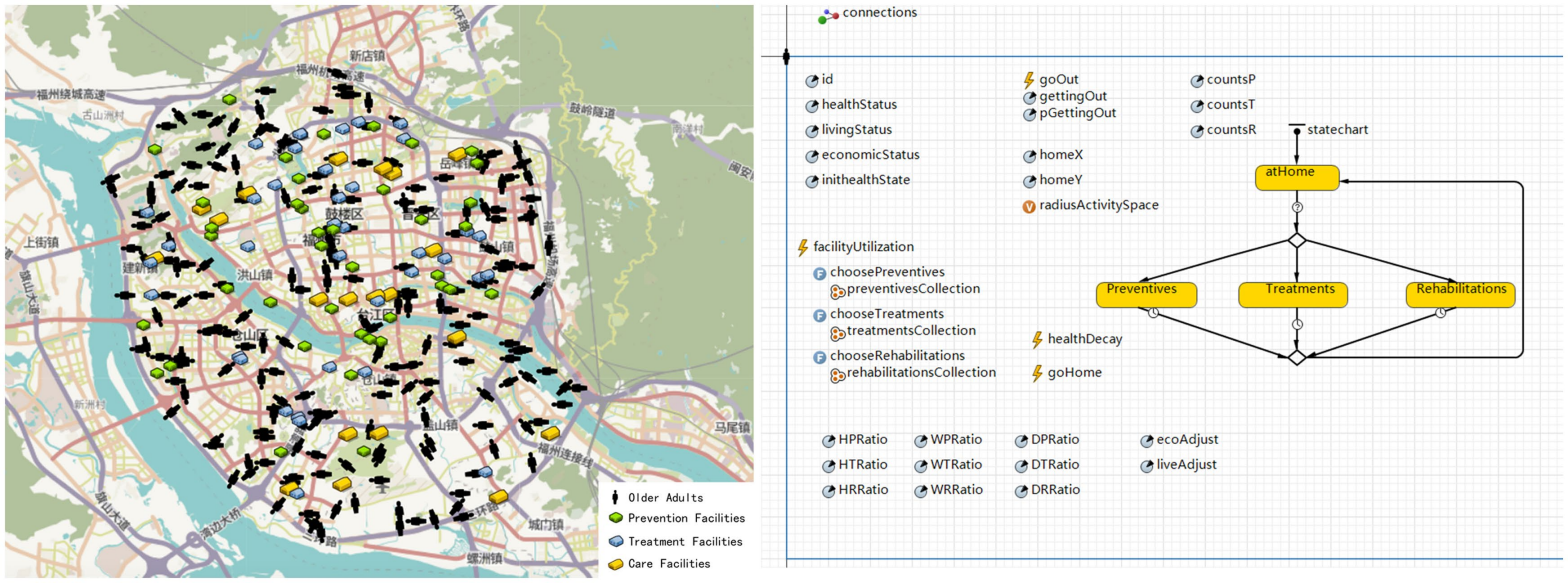
Model construction in AnyLogic GIS simulation environment. The base map and icons are all built-in components of the AnyLogic software.

The model environment encompasses the physical and spatial context necessary for the movement of older adults and their utilization of health facilities (Figure 4). It covers the entire urban area inside the Third Ring Road in Fuzhou’s main urban zone, incorporating a real-world road network and the actual geographic distribution of health facilities based on Point of Interest (POI) data.

##### 3.1.2.2 Agents and scales

Within this environment, the model incorporates two main types of agents: older adults and health facilities. A total of 200 older adult agents are randomly generated within the study area. Each is assigned initial attributes, such as health status, economic status, and living status, based on proportions derived from Fuzhou census data and calibrated through questionnaire surveys (as summarized in Table 1). Economic status is proxied by household vehicle value, a durable good widely recognized as a proxy for wealth in survey-based studies due to its comparability and data availability (49). Value thresholds correspond to established market segments (50). For example, vehicles valued below ¥100,000 represent low-income groups (entry-level vehicles), those between ¥100,000 and ¥300,000 represent middle-income (mainstream family vehicles), and those above ¥300,000 indicate high-income households (premium vehicles). These agents navigate the real road network to make daily travel and facility choices, with their health status updating dynamically after facility visits.

**Table 1:** Attributes and initialization of older adult agents.

Attribute	State	Description	Proportion
Health Status	healthy	Basically healthy	0.8
	weak	Unhealthy but self-care capable	0.16
	disabled	Disabled requiring assistance	0.04
Living Status	with spouse	with spouse	0.7
	with children	with children	0.29
	alone	alone	0.01
Economic Status	low	Household vehicle value < ¥100 k	0.69
	mid	Household vehicle value ¥100 k-300 k	0.26
	high	Household vehicle value ≥ ¥300 k	0.05

In addition, the model includes 100 facility agents generated from real POI locations, distributed as 40 prevention, 40 treatment, and 20 long-term care facilities, reflecting actual urban service ratios. Each type of facility is designed to provide distinct health benefits.

The model operates at a daily time step over a simulation period of 500 days, capturing the travel behavior and facility usage patterns of the older adult agents.

### 3.2 Design concepts

#### 3.2.1 Individual decision making

The decision-making processes for older adult agents in this model were designed based on empirical data obtained from questionnaires, with a primary focus on travel behavior. An analysis of 326 valid questionnaires collected from the main urban area of Fuzhou revealed that the health status of older adults exerts a significant gradient effect on both mobility and facility selection preferences. Statistical analyses—including the Kruskal-Wallis test, multivariate analysis of variance (MANOVA), and Dunn’s *post hoc* test—confirmed that health status significantly influences mobility capacity (Kruskal-Wallis test: *p* < 0.001). Specifically, healthier agents reported higher outing frequency (*M* = 4.69 days per week [Note: *M* denotes mean]) and broader travel ranges (*M* = 5.12 km), compared to those in the weak group (frequency *M* = 3.55 days, range *M* = 3.78 km) and the disabled group (frequency *M* = 2.01 days, range *M* = 1.02 km).

Based on these findings, the agents’ daily travel behavior is modeled according to their health status: healthy agents have a 70% probability of undertaking outings within a 5 km radius, weak agents have a 50% probability within 3 km, and disabled agents have a 30% probability within 1 km. When an outing is triggered, the agent first searches for available facilities within its activity radius and selects one at random. If no facilities are available within the radius, a secondary decision is invoked, with 50% probability, the agent will travel to the nearest facility outside the radius, otherwise, the outing is canceled.

#### 3.2.2 Interactions

Based on empirical questionnaire data and existing literature, this study formulates agent interactions centered around the utilization of health facilities by older adults. The interactive process is defined as the use of these assistive facilities, with behavioral rules informed by evidence-based influences of health status, economic standing, and living arrangements on older adults’ preferences and demands for health resources. As summarized in Table 2, insights derived from literature review directly guide the model’s rule design. For instance, healthier older adults tend to prioritize preventive activities such as autonomous exercise and social engagement (51, 52); those with chronic conditions (categorized as “weak”) show greater dependence on treatment facilities, consistent with patterns observed in chronic care management (53, 54); while disabled older adults exhibit higher reliance on long-term care resources, reflecting necessities for sustained support (55, 56).

**Table 2:** Correspondence between influencing factors and agent-based decision rules for health resource selection.

Attributes	Hierarchy of individual attributes	Potential model rules	References
Health status	Healthy	Healthy older adults prioritize prevention through exercise and social activities, leading to higher selection weights for preventive facilities.	(52)
	Weak	Chronically ill (weak) older adults prioritize treatment, reflecting medical reliance and resulting in higher selection weights for treatment facilities.	(53, 62)
	Disabled	Disabled older adults rely on recuperative care, resulting in higher selection weights for long-term care facilities.	(54–56)
Economic status	High	High-income older adults prioritize quality-of-life and long-term health management. Their financial capacity enables access to premium care options, resulting in higher selection weights for long-term care facilities.	(63)
	Mid	Middle-income older adults exhibit intermediate behavioral tendencies, often balancing prevention and treatment. This aligns with the sociological concept of “central tendency” in mid-tier groups, and therefore requires no additional model adjustments.	(64, 65)
	Low	Low-income older adults, due to limited financial capacity, rely more on low-cost or free preventive facilities such as community parks. This leads to higher selection weights for such resources, yet also correlates with accelerated health deterioration.	(66)
Living conditions	With Spouse	Older adults cohabiting with spouses exhibit comparatively higher selection weights for prevention resources.	(67)
	With Children	Older adults cohabiting with children exhibit comparatively higher selection weights for treatment resources.	(68)
	Alone	Solo-living older adults face deficits in ​immediate support, consequently elevating ​health deterioration risks​ with ​accelerated health decline rates.	(69)

These trends are corroborated by questionnaire findings, which confirm significant gradients in facility usage across health categories: healthier individuals prefer prevention facilities (mean usage frequency *M* = 4.69), those who are weak favor treatment facilities (*M* = 4.55), and disabled individuals predominantly use care facilities (*M* = 4.52), with MANOVA confirming statistically significant differences (*F* = 128.18, *p* < 0.001). Accordingly, the model operationalizes these patterns by dynamically assigning the facility-selection weights from Table 3 based on each agent’s current health status at every time step.

**Table 3:** Baseline facility preference weights by health status.

Current health status	Prevention weight	Treatment weight	Care weight
Healthy	0.6	0.2	0.2
Weak	0.3	0.5	0.2
Disabled	0.1	0.3	0.6

#### 3.2.3 Emergence

The emergence process within the model is characterized by dynamic changes in the health status of older adult agents, represented by a continuous variable H bounded between 0 (complete unhealthiness) and 1 (optimal health) (28), which is calibrated against the older adult health assessment system of the Fuzhou Population Census following China’s Seventh National Population Census protocol (39, 57). This system classifies health status through a unified self-reported health question combined with Activities of Daily Living (ADL) criteria, with the model’s three health states corresponding precisely to census classifications: the “healthy” state (H ∈ [0.7, 1.0]) represents individuals with full functional independence requiring no help in core ADLs; the “weak” state (H ∈ [0.3, 0.7]) corresponds to those with mild to moderate impairment requiring assistance with 1–2 ADLs; and the “disabled” state (H ∈ [0.0, 0.3]) signifies severe functional limitation or dependency requiring help with 3 or more ADLs. Health updating follows a discrete-time process influenced by natural decline and facility-based improvements, formalized as:


$$H_{t+1}=H_{t}\times (1-\alpha_{\mathrm{HS}}-\beta -\gamma )+H_{t}\times (1+\delta_{\text{type}})$$ (1)

Here (Equation 1), H_t_ denotes the health value at day t; α_HS_ represents the baseline health decay rate, stratified by health status; β and γ capture additional decay associated with solo living and low economic status, respectively; and δ_type_ indicates the health gain coefficient specific to each type of health facility (prevention, treatment, or long-term care). All health values are clipped to the interval [0, 1] to enforce biological plausibility. Specific parameter values, including decay rates and facility effect coefficients, are provided in Table 4.

**Table 4:** Key parameter values for health decay and facility gain functions.

Parameter Name	Value	Type	Description
healthyDecayRate	0.006	double	Natural decay rate for healthy older adults
weakDecayRate	0.009	double	Natural decay rate for weak (self-care capable) older adults
disabledDecayRate	0.012	double	Natural decay rate for disabled (requiring assistance) older adults
aloneDecayRate	0.001	double	Additional health risk for solo-living older adults
lowEconomicDecay	0.001	double	Additional health risk due to low economic status constraints
preHealthIncreaseRatio	0.001	double	Health effect coefficient for prevention facility usage
treHealthIncreaseRatio	0.003	double	Health effect coefficient for treatment facility usage
rehHealthIncreaseRatio	0.002	double	Health effect coefficient for care facility usage

To evaluate equity implications emerging from agent interactions, a weighted Gini coefficient is applied across three core health equity indicators, improving upon conventional two-group inequality measures (58), formalized as:


$$G=\frac{\sum_{1\leq i<j\leq 3}w_{i}w_{j}|x_{i}-x_{j}|}{2\sum_{i=1}^{3}w_{i}x_{i}}$$ (2)

This metric incorporates observations x₁, x₂, x₃, weighted by w₁, w₂, w₃, derived from demographic proportions reported in the Fuzhou population census, enabling a finer-grained assessment of distributional fairness in health outcomes (Equation 2). The observed values (x₁, x₂, x₃) represent the average health values of the model outputs for subpopulations of agents with different attributes. These subpopulations are categorized based on three types of individual attributes: initial health status (x₁-healthy, x₂-weak, x₃-disabled), income level (x₁-high, x₂-mid, x₃-low), and living status (x₁-with spouse, x₂-with children, x₃-alone). The calculation method is identical for each attribute and is performed separately. The weights (w₁, w₂, w₃) are derived from the actual census data of the main urban area of Fuzhou City, as shown in Table 1. They represent the actual proportion of each of the aforementioned subpopulations within the total population of the main urban area of Fuzhou City.

### 3.3 Design details

#### 3.3.1 Initialization

**Figure 5:**
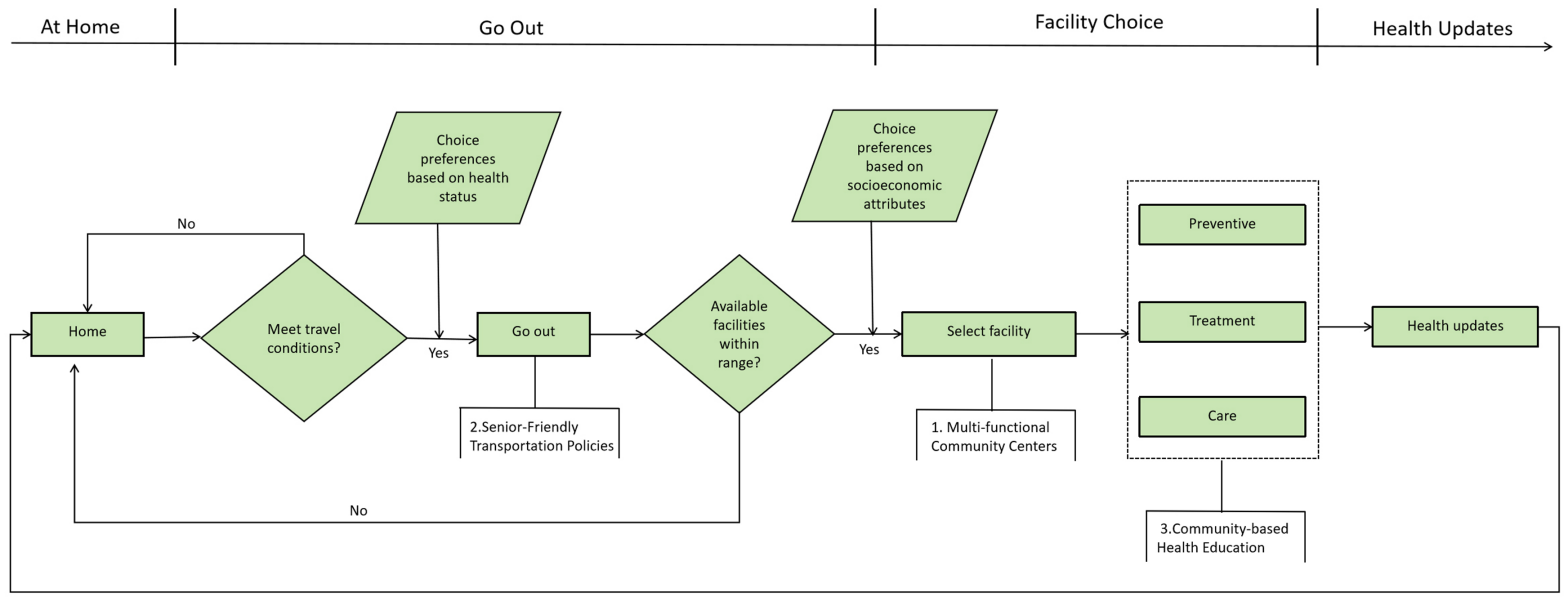
Flowchart of the agent-based decision process for travel and facility utilization.

During the initialization phase, older adult agents are randomly distributed across the area within Fuzhou’s Third Ring Road. Each agent follows a daily cycle that includes deciding whether to go out, selecting and traveling to a facility, returning home, and updating their health status accordingly. The travel and facility selection process is guided by the logic depicted in Figure 5.

#### 3.3.2 Submodels

Building upon this baseline model, four intervention scenarios are implemented as submodels to evaluate the effects of policy interventions across spatial, social, and familial dimensions on health outcomes among older adults.

##### 3.3.2.1 Scenario 1: multi-functional community centers

In line with China’s “aging-in-place” agenda, this scenario scales up Fuzhou’s flagship “Older-Adult Canteen + School” and “Community-Embedded Older-Adult Care” programs. Ten strategically chosen community centers are upgraded into one-stop health hubs, represented in the model by ten new facility agents whose placement algorithm priorities the largest service-coverage gaps. Each hub bundles daycare, recuperative care, preventive screenings and socialization spaces, delivering compound health benefits that exceed the sum of their parts.

##### 3.3.2.2 Scenario 2: senior-friendly transportation policies

To dismantle the “last-mile” mobility barrier, this intervention deploys a comprehensive senior-friendly transportation package, such as free or low-fare dedicated health-transit lines, barrier-free bus retrofits (low-floor boarding, priority seating, audible/visual announcements), community micro-circulation shuttles with flexible routing and on-demand stops, and pedestrian upgrades, designed to create a low-stress travel chain from home to facility. In the agent-based model, these improvements are operationalized by increasing two core parameters: the daily outing probability (*pGettingOut*) and the activity radius (*radiusActivitySpace*). By explicitly translating the multidimensional “last-mile” concept into these measurable behavioral rules, S2 enhances spatial accessibility and facilitates the utilization of previously unreachable health facilities, enabling a precise assessment of its policy impact.

##### 3.3.2.3 Scenario 3: community-based health education

This scenario deploys a multi-format curriculum, such as on-site workshops, short-form videos and family-oriented seminars, aimed simultaneously at older adults and their adult children. The content stresses chronic disease self-management and nurtures intergenerational support norms. Rather than adding new facilities, the model re-weights existing ones. For example, community centers offering recurrent health literacy sessions receive higher selection probabilities, translating gains in awareness into measurable upticks in preventive care uptake.

##### 3.3.2.4 Scenario 4: comprehensive interventions

This scenario overlays the three preceding strategies (S1, S2, S3) through a rule-based parametric superposition. Specifically, for parameters of the same category present in multiple scenarios, the maximum value is selected (e.g., *pGettingOut*, *radiusActivitySpace*). The remaining parameters and model adjustments, such as the addition of new facility agents from S1, are cumulatively incorporated.

All resulting parameter adjustments are documented in full detail in Table 5, ensuring transparent replication of the integrated model run. Following multiple simulation runs with exclusion of the initial 60-day model burn-in period, results from the ABM were systematically evaluated across two core dimensions: (1)Health status, quantified by the health indicator H (range: 0–1; 0 = complete unhealthiness, 1 = optimal health); and (2) Health equity, measured via Gini coefficients (range: 0–1; 0 = perfect equity) across three socioeconomic status: initial health status (Healthy/Weak/Disabled), economic status (Low/Mid/High), and residential conditions (With spouse/With children/Alone). The results synthesize the impacts of four interventions—S1 (Multi-functional Community Centers), S2 (Senior-Friendly Transportation Policies), S3 (Community-based Health Education), and S4 (Comprehensive Intervention)—on health outcomes and equitable resource access for older adults in Fuzhou’s urban core.

**Table 5:** Intervention scenario parameter adjustments.

Scenario	Baseline parameters	Intervention parameters
S1	100 points	+10 comprehensive older adults health resource pointsnewhealthincreaseRatio-0.05
S2	Health status-pGettingOut /radiusActivitySpace Healthy-5 km/0.7 Weak-3 km/0.5 Disabled-1 km/0.3	Health status -pGettingOut/radiusActivitySpace Healthy-10 km/0.8 Weak-8 km/0.7 Disabled-3 km/0.4
S3	liveAdjust-0.1 Health statues -pGettingOut Healthy-0.7 Weak-3 km/0.5 Disabled-1 km/0.3	liveAdjust-0.2 Health statues -pGettingOut Healthy-0.75 Weak-0.6 Disabled-0.35
S4	Parametric superposition of Scenarios 1–3	

## 4 Results

### 4.1 Spatial accessibility: the primary driver of population health gains

**Figure 6:**
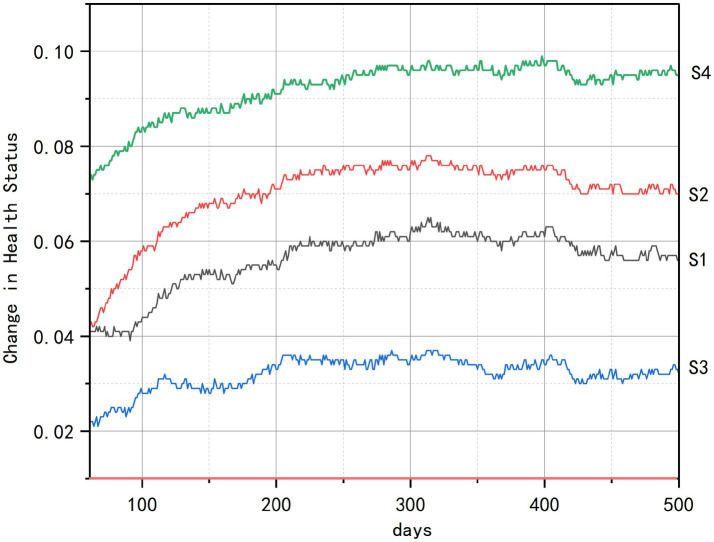
Changes in average health status of older adults.

As evidenced in Figure 6, all intervention scenarios generated significant health improvements relative to the baseline (0.0), with spatial accessibility emerging as the paramount determinant of health gains. The comprehensive intervention (S4) achieved the most substantial overall improvement by synergistically integrating multidimensional strategies. Among the standalone interventions, the senior-friendly transportation policy (S2) demonstrated the most rapid health improvement, distinguished by the steepest ascent in its curve during the initial simulation period (approximately days 60–100).

These strong positive results, combined with consistent benefits across the older adult population, demonstrate the critical importance of improving spatial accessibility to overcome ‘last-mile’ mobility barriers. Community center repurposing (S1) ranked second in effectiveness by expanding daily health maintenance opportunities, though its impact remained constrained by service radius limitations that restricted usage frequency. In contrast, the community-based health education (S3) exhibited the weakest momentum and earliest stagnation plateau, reflecting significant vulnerability to individual variability in knowledge conversion efficiency driven by uncontrolled factors such as educational disparities. The consistent efficacy hierarchy observed (S4 > S2 > S1 > S3), further reinforces spatial accessibility as the foundational catalyst for health optimization.

### 4.2 Multidimensional health equity: differential intervention impacts across socioeconomic status

**Figure 7:**
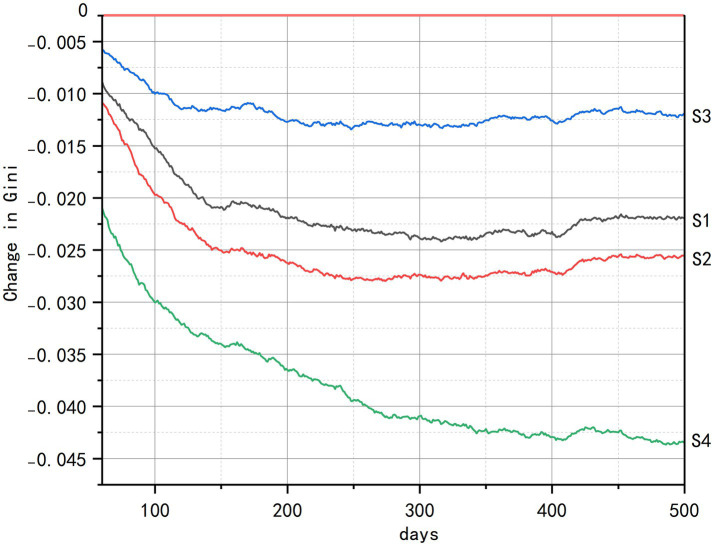
Changes in health Gini coefficients by initial health status.

**Figure 8:**
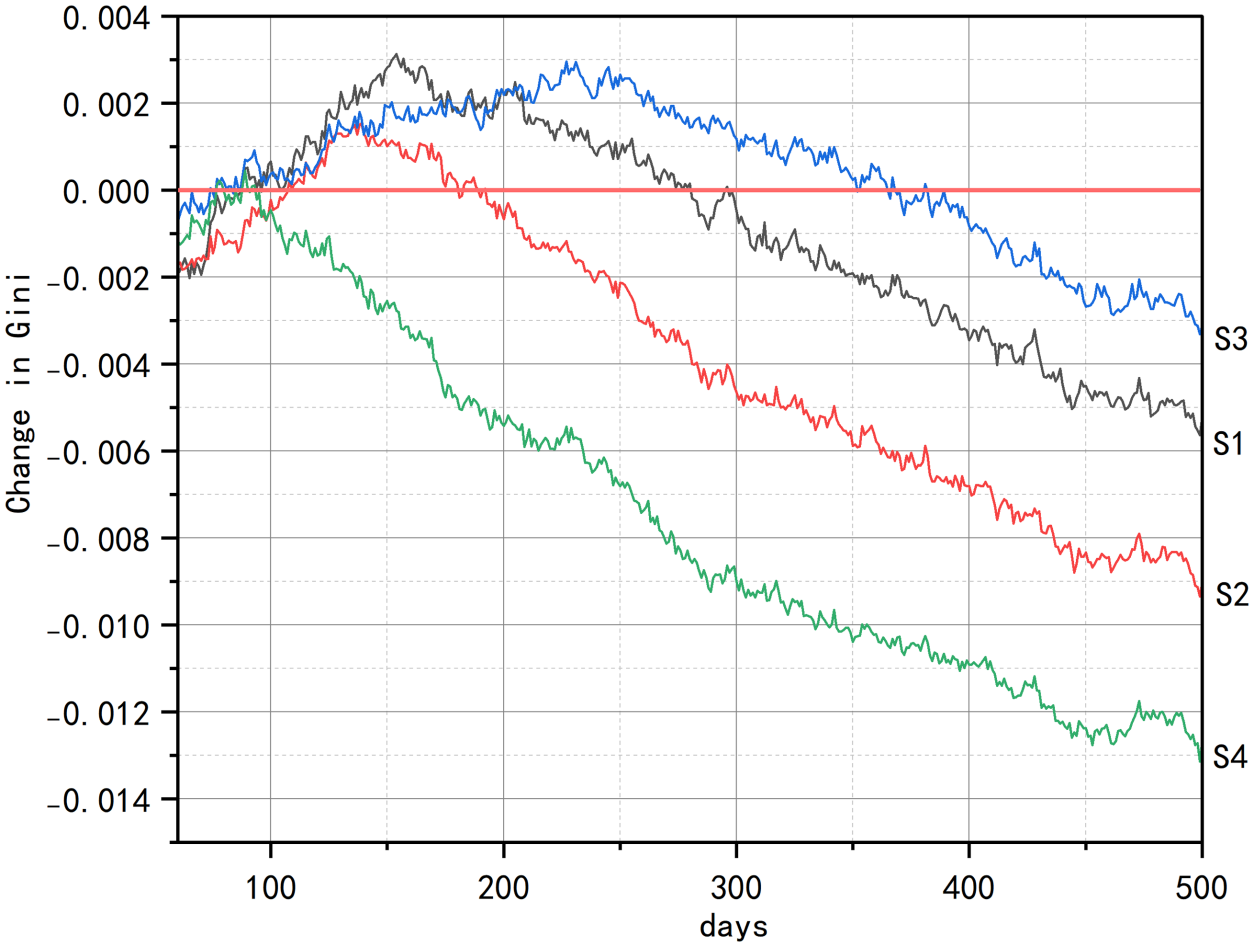
Changes in health Gini coefficients by economic status.

**Figure 9:**
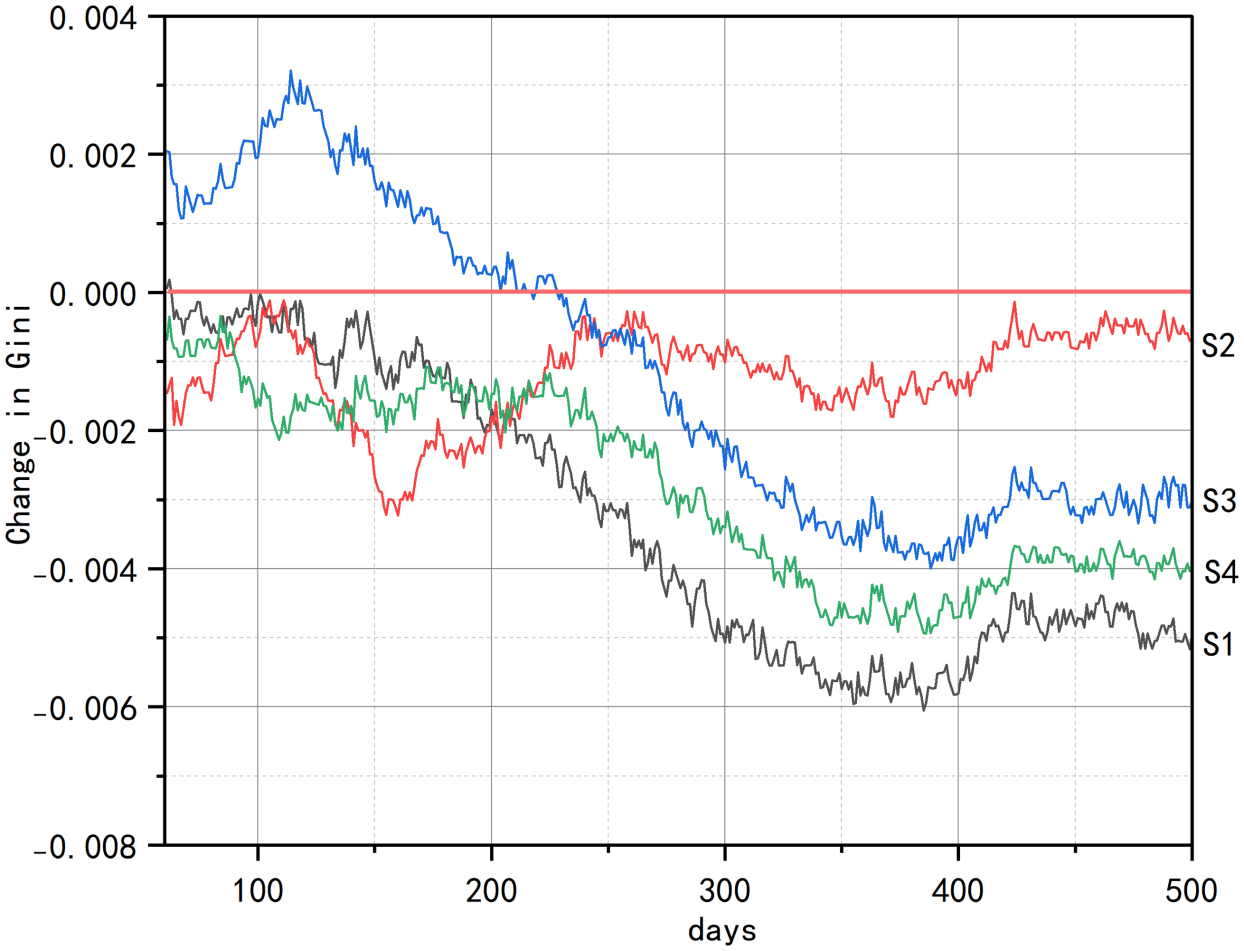
Changes in health Gini coefficients by living status.

Analysis of health equity impacts reveals consistent intervention hierarchies but context-dependent efficacy across socioeconomic dimensions, as quantified by Gini coefficient index (Figures 7–9). Across all strata, such as initial health status, economic status, and living status, comprehensive interventions (S4) demonstrated sustained equity improvements, achieving the largest Gini coefficient reductions through synergistic integration of spatial, cognitive, and social components. The senior-friendly transportation policies (S2) consistently ranked second in efficacy, generating rapid initial equity gains across health and economic subgroups by overcoming spatial accessibility barriers.

Across subgroups defined by baseline health status (Figure 7), the integrated intervention (S4) leveraged multidimensional coupling to produce a gradual yet durable reconstruction of equity. In contrast, senior-friendly transportation policies (S2) that rely primarily on improving spatial accessibility exhibited diminishing marginal returns over time. Critically, although the community-based health education (S3) nominally offered opportunities for participation, the persistent mobility constraints of disabled older adults meant that the actual benefits they derived were minimal.

For groups differentiated by economic status (Figure 8), all interventions triggered a transient deterioration in equity at the outset. This occurred because policy dividends were first captured by population segments with stronger resource-acquisition capacity, while disadvantaged groups, constrained by delayed penetration of resources into space, barriers to cognitive translation, and information asymmetry, experienced postponed gains, thereby temporarily widening gaps in access to health resources. Following the initial implementation shock, senior-friendly transportation policies (S2) emerged as the most effective standalone intervention. By physically expanding older adults’ activity spaces through sidewalk upgrades, strategically placed rest nodes, and dedicated senior transit loops, this approach directly dismantled concrete access barriers to health resources. The community-based health education (S3) eventually contributed to health gains, though its impact required an extended maturation period for knowledge translation and intergenerational support networks to develop fully. Most critically, the multi-functional community centers (S1) carried significant implementation risks. When deployment prioritized facility quantity over precise spatial targeting- specifically, failing to locate hubs within identified service deserts-these centers were disproportionately accessed by higher-income users. This “static hardware deployment” approach consequently amplified pre-existing inequities rather than alleviating them, demonstrating that infrastructure placement without equity-focused siting criteria can undermine intervention goals.

Among populations distinguished by living status (Figure 9), the multi-functional community centers (S1) became the single most effective intervention by directly compensating for the absence of family support—particularly for older adults living alone. This was achieved by expanding the physical supply of health resources, prioritizing the filling of service-coverage gaps, and providing day-care services. In comparison, the integrated intervention (S4) underperformed S1 overall, mainly because of unstable effects from spatial mobility enhancement (S2) and the behavior-change dependency of health education (S3), which jointly weakened synergy. The senior-friendly transportation policies (S2) displayed a brief but pronounced initial impact, yet their effectiveness declined continuously thereafter. More importantly, senior-friendly transportation policies (S2) failed to counter the inherent risk of accelerated health decline in isolated living environments such as solitary residence, and inadvertently intensified health inequities tied to differential living status.

### 4.3 Model robustness and sensitivity analysis

**Figure 10:**
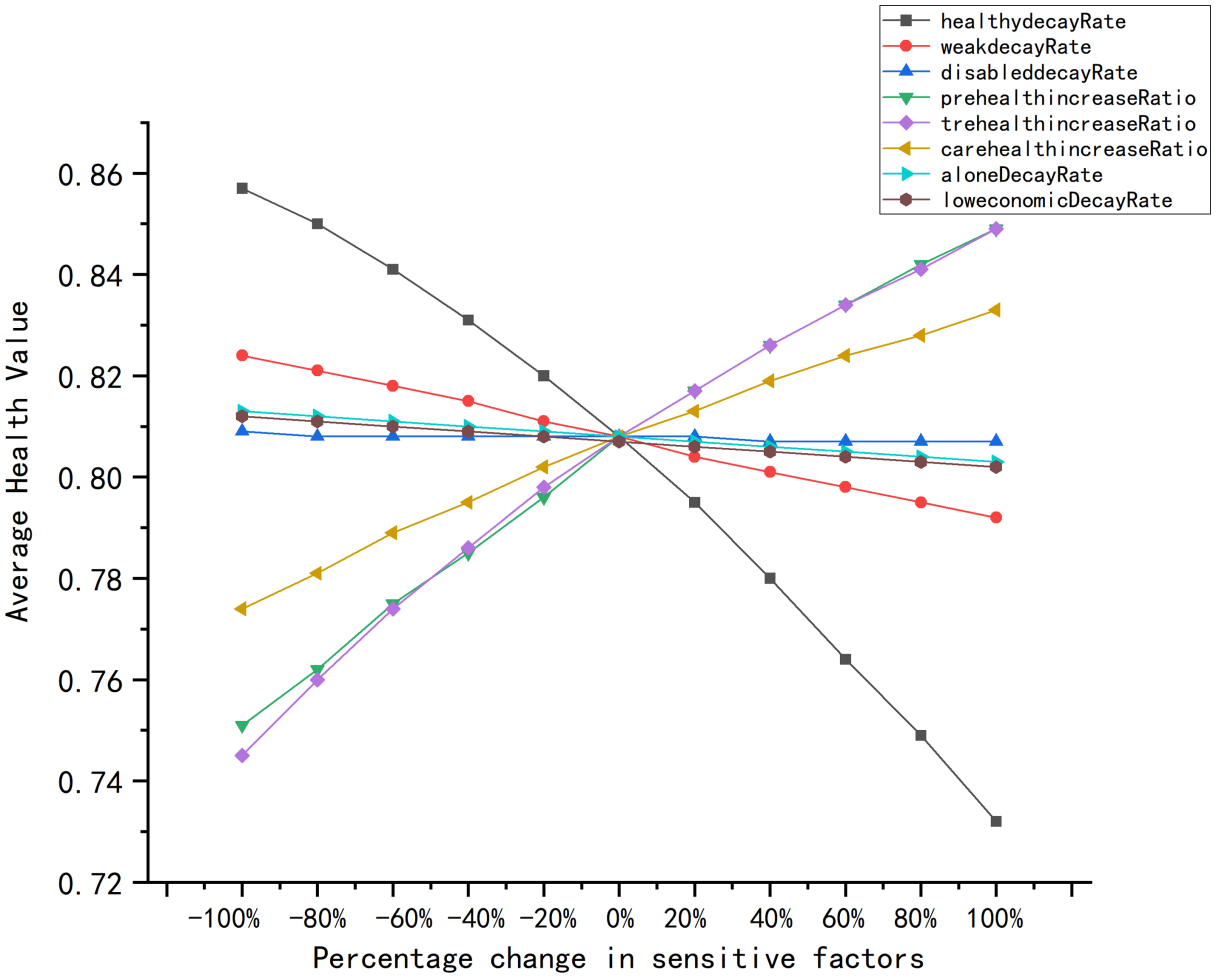
Sensitivity analysis.

This study established the model’s reliability for intervention assessment through rigorous robustness verification and sensitivity analysis. Univariate sensitivity testing (Figure 10) conducted across 100 independent simulation trials confirmed exceptional stability against input perturbations, with consistent predictive performance maintained throughout parameter variations. The smooth, continuous response curves, which were devoid of abrupt jumps or anomalous oscillations, demonstrated resilient output behavior under operational disturbances. Core parameters including health decay rates and preventive resource efficacy consistently aligned with theoretical expectations, validating the model’s mechanistic logic. Notably, while most parameters exhibited moderate sensitivity, select variables (e.g., decay rates for disabled subgroups) showed minimal responsiveness near baseline values, indicating strong tolerance to minor fluctuations. This balanced sensitivity profile, which avoids both excessive dependence on individual parameters and loss of critical effect detection, ultimately affirms the framework’s capacity for reliable policy extrapolation under uncertainty. The confirmed adaptability positions this ABM as a robust quantitative tool for forecasting multi-scenario health outcomes and optimizing aging-resource allocation strategies.

## 5 Discussion

The intervention scenarios evaluated in this study are strategically aligned with China’s active aging policy framework. Scenario S2 (Senior-Friendly Transportation) directly operationalizes the national mandate from the “14th Five-Year Plan for the Aging Cause and Older Adults Care System” to enhance age-adapted transformation of urban transportation (38). Similarly, Scenario S1 (Multi-functional Community Centers) implements the national “embedded older adults care” policy that promotes integrated community service hubs (2). Similarly, Scenario S3 (Community-based Health Education) is explicitly anchored in the “Healthy China 2030” blueprint and the “14th Five-Year Plan for Healthy Aging,” both of which prioritize health literacy promotion and chronic-disease self-management at the grassroots level by translating the national requirement for “community-based health education rooms and lecture series” into a scalable intervention (3, 38). This deliberate policy grounding ensures that our modeling exercise addresses real-world priorities.

In this context, the ABM simulation approach provides critical advantages for evidence-based policy formation. By creating a controlled “policy laboratory,” this study enable low-risk testing of intervention concepts before committing substantial public resources. The model identifies both leverage points and potential unintended consequences, such as the risk of S1 reinforcing spatial inequities, that might only emerge after costly implementation. This preemptive screening capacity is particularly valuable for aging cities like Fuzhou that face urgent resource allocation decisions amid demographic pressures.

The analysis demonstrates that transportation accessibility serves as the primary driver of health improvement, with S2 producing the most rapid gains by directly expanding older adults’ “mobility circles.” This finding highlights the critical importance of addressing the spatial dimension of the “last-mile” problem as a foundation for health equity in high-density urban environments. It is noteworthy that while this study focused on Fuzhou’s densely populated urban core, the fundamental mechanism through which transportation accessibility enables health resource utilization likely has broader applicability across diverse urban settings.

The relatively modest outcomes observed in the health education scenario (S3) highlight the significant influence of cognitive and socioeconomic factors in shaping the success of behavioral interventions. This finding reinforces the importance of situating educational initiatives within easily reachable physical settings and established social support systems—an approach consistent with China’s integrated care model, though one that demands adaptation to local conditions for effective implementation (59–61).

## 6 Conclusion

This study establishes that spatial accessibility constitutes the most critical determinant of health outcomes and equity for older adults in high-density urban cores like Fuzhou. Through an agent-based model capturing dynamic interactions across prevention-treatment-care resources, this study demonstrates that comprehensive interventions (S4), integrating spatial, transportation, and social dimensions, deliver the most significant and robust improvements. Such multidimensional strategies substantially elevate population-wide health while systematically dismantling barriers driven by “mobility circle collapse” among disadvantaged subgroups, achieving measurable equity gains across health and economic strata. However, their efficacy remains constrained in addressing residential inequities due to synergy limitations. Senior-friendly transportation policies (S2) emerge as potent catalysts for rapid health and equity improvements across health/economic groups, yet paradoxically exacerbate disparities among residential subgroups. While multi-functional community centers (S1) enhances health maintenance opportunities, its impact remains bounded by spatial distribution imbalances that risk solidifying disparities in underserved areas. Community-based health education (S3) yields minimal returns, underscoring how cognitive capacity, socioeconomic status, and family support act as persistent filters for knowledge conversion.

This study establishes agent-based modeling as a valuable tool for bridging national policy directives with local implementation planning. By translating policy concepts into computationally testable scenarios, this research provides a methodology for simulating intervention impacts across diverse urban contexts. Future research should expand this paradigm by incorporating multi-agent interactions with family and community networks, integrating cost-effectiveness analysis, and validating findings across cities with varying geographic and socioeconomic characteristics. Such efforts will further strengthen the evidence base for designing targeted, effective policies to address the complex challenges of population aging.

## Data Availability

The raw data supporting the conclusions of this article will be made available by the authors, without undue reservation.
