# Supplementary material for: Agent-based modeling of health resources for older adults: accessibility, equity, and last-mile solutions in Fuzhou, China
Source: Front Public Health. 2025 Nov 24;13:1698911. doi: 10.3389/fpubh.2025.1698911 (PMC12683718; doi:10.3389/fpubh.2025.1698911)
Supplement: Supplementary file 1 [file Table_1.pdf]

# Appendix

## A1. Questionnaire

| Section | Item | Question | Response Options |
|---|---|---|---|
| **I. Basic Info** | 1 | Gender | M / F |
| | 2 | Age | 60-64 / 65-69 / 70-74 / 75-79 / ⩾80 |
| | 3 | District | Gulou / Taijiang / Cangshan / Jin'an / Mawei / Minhou |
| **II. Health & Activities** | 4 | Health status (past 6 months) | □ Generally healthy (No chronic diseases; able to perform daily activities independently)<br>□ Unhealthy, but self-sufficient (Weak, Minor manageable chronic diseases requiring long-term medication/regular medical visits)<br>□Unhealthy, not self-sufficient (disabled, Major illness/mobility impairment requiring long-term personal care) |
| | 5 | Weekly outdoor days | 0 / 1-2 / 3-4 / ≥5 |
| | 6 | Max. travel distance | <1km / 1-3km / 3-5km / >5km |
| **III. Socioeconomics** | 7 | Monthly income (CNY) | <3,000 / 3,000-5,000 / 5,000-8,000 / >8,000 |
| | 8 | Living arrangement | Alone / With spouse / With children / Other:_____ |
| **IV. Facility Use** | 9a | *Preventive facilities*:<br>Frequency (1-5)[1]<br>Purpose | 1/2/3/4/5<br><br>Fitness/Social/Leisure |
| | 9b | *Treatment facilities*:<br>Frequency (1-5)[1]<br>Purpose | 1/2/3/4/5<br><br>Chronic/Acute/Other |
| | 9c | *Long-term Care facilities*:<br>Frequency (1-5)[1]<br>Purpose | 1/2/3/4/5<br><br>Daily care/ Rehab/ Therapy |
| | 10 | Choice factors | Proximity/Cost/Quality/Family/Health needs/Other:_____ |
| **V. Feedback** | 11 | Priority improvement | Accessibility/Free checkups/Activities/Home care/Other:_____ |
| | 12 | Suggestions | __________ |

## A2. Model Parameters

| Class | Parameter Name | Type | **Description** |
|---|---|---|---|
| Main | healthydecayRate | double | Natural decay rate for **healthy** older adults |
| | weakdecayRate | double | Natural decay rate for **weak**(self-care capable) older adults |
| | disableddecayRate | double | Natural decay rate for **disabled**(requiring assistance) older adults |
| | aloneDecayRate | double | Additional health risk for **solo-living** older adults |
| | loweconomicDecay | double | Additional health risk due to **low economic status** constraints |
| | prehealthincreaseRatio | double | Health effect coefficient for *prevention* **facility usage** |
| | trehealthincreaseRatio | double | Health effect coefficient for *treatment* **facility usage** |
| | carehealthincreaseRatio | double | Health effect coefficient for *care* **facility usage** |
| Older Adult | id | int | Unique agent identifier for older adults |
| | homeX | double | Longitude coordinate of older adults agent's residence |
| | homeY | double | Latitude coordinate of older adults agent's residence |
| | healthStatus | double | Current dynamic health value |
| | inithealthState | int | Initial health status (healthy/weak/disabled) |
| | livingStatus | int | Residential condition (with spouse/with children/alone) |
| | economicStatus | int | Economic status (low/mid/high) |
| | pGettingOut | double | Daily outing probability |
| | radiusActivitySpace | double | Maximum activity radius for travel |
| | HPRatio | double | *Prevention* facility selection probability for **healthy** older adults |
| | HTRatio | double | *Treatment* facility selection probability for **healthy** older adults |
| | HCRatio | double | *Care* facility selection probability for **healthy** older adults |
| | WPRatio | double | *Prevention* facility selection probability for **weak** older adults |
| | WTRatio | double | *Treatment* facility selection probability for **weak** older adults |
| | WCRatio | double | *Care* facility selection probability for **weak** older adults |
| | DPRatio | double | *Prevention* facility selection probability for **disabled** older adults |
| | DTRatio | double | *Treatment* facility selection probability for **disabled** older adults |
| | DCRatio | double | *Care* facility selection probability for **disabled** older adults |
| | ecoAdjust | double | Economic status adjustment weight for facility selection |
| | liveAdjust | double | Residential condition adjustment weight for facility selection |
| Prevention Facility | longitude | double | Longitude coordinate of *prevention* facility |
| | latitude | double | Latitude coordinate of *prevention* facility |
| Treatment Facility | longitude | double | Longitude coordinate of *treatment* facility |
| | latitude | double | Latitude coordinate of *treatment* facility |
| Care Facility | longitude | double | Longitude coordinate of *care* facility |
| | latitude | double | Latitude coordinate of *care* facility |
